# Admission screening and cohort care decrease carbapenem resistant enterobacteriaceae in Vietnamese pediatric ICU’s

**DOI:** 10.1186/s13756-021-00994-9

**Published:** 2021-08-30

**Authors:** K. Garpvall, V. Duong, S. Linnros, T. N. Quốc, D. Mucchiano, S. Modeen, L. Lagercrantz, A. Edman, N. K. Le, T. Huong, N. T. B. Hoang, H. T. Le, D. TK. Khu, D. M. Tran, P. H. Phuc, H. Hanberger, L. Olson, M. Larsson

**Affiliations:** 1 Department of Global Public Health, Karolinska Institutet, Tomtebodavägen 18B 4fl, 171 77 Stockholm, Sweden; 2 Vietnam National Children’s Hospital, 18 ngõ 879 Đường La Thành, Láng Thượng, Đống Đa, Hà Nội, Vietnam; 3 Training and Research Academic Collaboration (TRAC), Sweden – Vietnam, 18 ngõ 879 Đường La Thành, Láng Thượng, Đống Đa, Hà Nội, Vietnam; 4 Research Institute for Child Health, 18 ngõ 879 Đường La Thành, Láng Thượng, Đống Đa, Hà Nội, Vietnam; 5 Department of Clinical Microbiology and Department of Clinical and Experimental Medicine, Linköping University, 581 83 Linköping, Sweden; 6 Department of Women’s and Children’s Health, Karolinska Institutet, Tomtebodavägen 18B 8fl, 171 77 Stockholm, Sweden

**Keywords:** Cohort care, Carbapenem resistant *Enterobacteriaceae*, Hospital acquired infections, Admission screening, Pediatric and neonatal care

## Abstract

**Objectives:**

To assess if admission screening for Carbapenem Resistant *Enterobacteriaceae* (CRE) and cohort care can reduce CRE acquisition (CRE colonization during hospital stay), Hospital Acquired Infections (HAI), hospital-stay, mortality, and costs in three Intensive Care Units (ICU’s) at the Vietnamese National Children’s Hospital.

**Method:**

CRE screening using rectal swabs and *ChromIDCarba*s elective culture at admission and if CRE negative, once weekly. Patients were treated in cohorts based on CRE colonization status.

**Results:**

CRE colonization at baseline point-prevalence screening was 76.9% (103/134). Of 941 CRE screened at admission, 337 (35.8%) were CREpos. 694 patients met inclusion criteria. The 244 patients CRE negative at admission and screened > 2 times were stratified in 8 similar size groups (periods), based on time of admission. CRE acquisition decreased significant (OR − 3.2, *p* < 0.005) from 90% in period 2 (highest) to 48% in period 8 (last period). Patients with CRE acquisition compared to no CRE acquisition had a significantly higher rate of culture confirmed HAI, n = 20 (14%) vs. n = 2 (2%), longer hospital stays, 3.26 vs. 2.37 weeks, and higher total treatment costs, 2852 vs. 2295 USD.

**Conclusion:**

Admission CRE screening and cohort care in pediatric ICU’s significantly decreased CRE acquisition, cases of HAI and duration of hospital-stay.

## Introduction

Antibiotic resistance is resulting in increased morbidity, mortality and healthcare costs [1]. In South East Asia, the situation is especially severe [2, 3]. Hospital acquired infections (HAI) with Carbapenem Resistant *Enterobacteriaceae* (CRE) are resistant to most antibiotics [4, 5] and very difficult to treat even with last resort antibiotics including tigecycline, gentamicin, amikacin and colistin [4, 6].

CRE colonization and HAI are common in Vietnamese intensive care units (ICU’s) [2,7,8]. CRE colonized patients with HAI has high mortality [7,9,10,6,9–12].

,^12^–^14^,^15^,^12^,^14^,

## Materials and methods

### Design

**Fig. 1:**
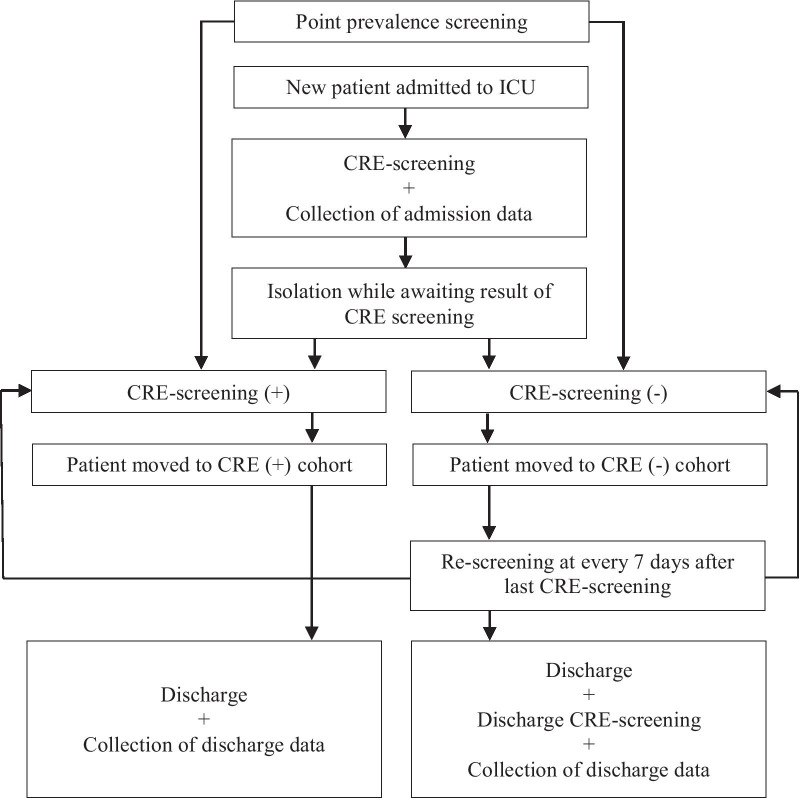
CRE screening and cohort care intervention flowchart

This was a prospective intervention cohort study with a quasi-experimental design at the Vietnamese National Children’s Hospital (VNCH) ICU’s It was performed to assess the effectiveness of CRE admission screening and cohort care on CRE acquisition and colonization, HAI, treatment outcome and costs. Implemented from 2018-03-20 to 2018–06-20 (Fig. 1). Conventional Infection Prevention and Control (IPC) measures were already in place in the ICU’s, including instructions for hand hygiene, availability of alcoholic hand rub, personal protective equipment, gloves and shoe protection, and an active antibiotic stewardship group. The project promoted and strengthened these IPC measures.

According to CRE colonization status in the baseline PPS in the ICU’s, patients were moved to CRE positive (CREpos) or CRE negative (CREneg) cohorts. Following the PPS, all new admissions were CRE screened and cohorted in a separate area for newly admitted patients with unknown colonization status. The fecal screening samples were transported to the microbiology lab and cultured. According to screening result, patients were moved to either the CREpos or CREneg cohort. The cohort care intervention started at the same time as the admission screening as it was considered unethical to not cohort patient once their CRE-status was known. Patients screening CREneg were re-screened every 7 days of ICU care and discharge screened if > 48 h after admission. All patients diagnosed with a HAI were isolated in a separate area with designated staff according to pre-existing ICU protocols. ICU staff implemented the intervention and collected patient data on paper forms in Vietnamese.

### Participants

#### Inclusion criteria

1. All patients admitted to the ICU’s.
2. For newborns admitted to the Neonatal Intensive Care Unit (NICU), CRE admission screening was performed at the earliest 24 h after birth (as detectable bacterial intestinal colonization is not established < 24 h).
3. Patients already admitted before the start of the intervention were screened and treated in cohorts according to CRE status but were not included in the intervention.

### Setting

The study was implemented at the VNCH’s 3 main pediatric ICU’s: the general Pediatric Intensive Care Unit (PICU); the Surgical Intensive Care Unit (SICU) and the NICU. In total, there were 176 beds (NICU n = 80, PICU n = 62, SICU n = 34), all typically occupied.There were up to 20 beds in the same room in the NICU, up to 10 in the PICU and 3–6 beds in the SICU. Each ward was divided into three sections: for newly arrived patients awaiting screening results; for patients screened CREneg and for patients screened CREpos.

### Bacterial culture

Fecal samples were collected using rectal swabs (*ESwabs: Copan, Brescia, Italy*,*ChromID Carba: Biomérieux, Marcy-l’Étoile, France*). Incubation time 18 h as per manufacturer’s instructions [16,

### Economic outcomes

Data on the cost of hospital stay was obtained from the hospital economic division after patient discharge. The total cost was assessed in relation to CRE colonization and HAI. CRE screening cost was estimated at 3 USD per patient / two screenings.

### Sample size

The sample size was based on admission CRE colonization rate of ~ 30% and at discharge ~ 80% [7,

### Statistics

The *primary outcome*,*Secondary outcomes*,*Microsoft Corp*) was used for preliminary data input. The *IBM SPSS Statistics software* (*version 22 IBM, CA*,*Free Statistics and Forecasting Software *[17] and *Social Science Statistics *[18]*.* *p*-value of < 0.05 was considered significant.

## Results

### Baseline CRE point prevalence survey

Baseline CRE PPS was performed on the 20th of March 2018 in the NICU: 21st in the SICU and 22nd in the PICU. 103/134 patients (76.9%) were CRE colonized: NICU 79.4% (54/68), SICU 60.7% (17/28) and PICU 84.2% (32/38) (Table 1).

**Table 1:** Point prevalence data

Variables	NICU N (%)	PICU N (%)	SICU N (%)	Total N (%)
CRE +	54 (79)	32 (84)	17 (60)	103 (77)
Crude mortality	15 (22)	13 (34)	6 (21)	34 (25)
Total	68 (100)	38 (100)	28 (100)	134 (100)
*Klebsiella pneumoniae*	43 (80)	16 (50)	7 (41)	66 (49)
*Escherichia coli*	33 (61)	20 (62)	2 (12)	55 (41)
KESC*	6 (11)	10 (31)	5 (29)	21 (16)
Other**	6 (11)	8 (25)	7 (41)	21 (16)
*Acinetobacter baumannii*	3 (6)	0	0	3 (2.2)
*Pseudomonas aeruginosa*	1 (2)	0	1	2 (1.5)

^*^KESC Klebsiella species., Enterobacter species, Serratia marcescens and Citrobacter species

^**^Other = culture growth, bacteria not identifiable

### Admission CRE screening

**Fig. 2:**
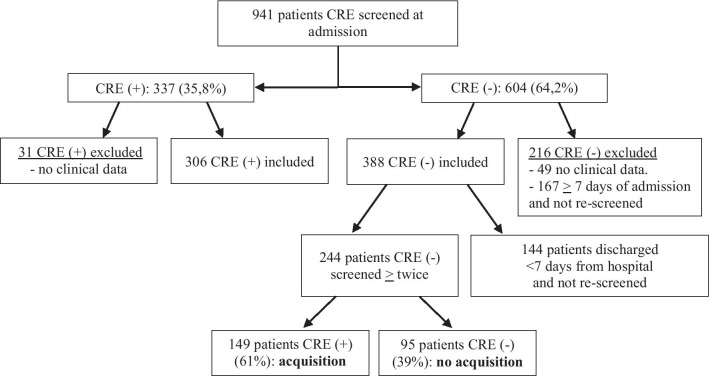
Flowchart for patient recruitment

A total of 941 patients were CRE screened at admission to the ICU’s. 337 (35.8%) were CRE colonized: NICU 39.6% (165/417), PICU 40.6% (91/224) and SICU 27,0% (81/300). Of the 694 patients (73.8%) that met the inclusion criteria, 306 (44.1%) were CREpos: NICU 46.7% (151/323), PICU 48.8% (84/172) and SICU 35.7% (71/199). Of the 247 patients excluded, 80 lacked clinical data: 31 (38.8%) CREpos; another 167 were CREneg and admitted > 7 days and not rescreened, hence acquisition status could not be determined. Of the 388 patients screened CREneg at admission and included, 244 (62.9%) were screened > 2 times and could be included in the acquisition assessment cohort. 144 (37.1%) of the CREneg patients were admitted < 7 days and not rescreened (Fig. 2).

There was a significant correlation between CRE colonization and HAI (*p* < 0.005) as well as HAI related mortality (*p* < 0.05) when compared to CREneg patients. CREpos patients had a significantly higher number of hospitals days and healthcare cost compared to CREneg patients, 23.4 days and 2568 USD vs. 8.1 days and 1351 USD, respectively (Table 2). For patients with a mortal outcome, the average duration of hospital stay was significantly longer for CREpos patients (27.6 days) vs. CREneg (8.8 days).

**Table 2:** General characteristics in groups as per CRE-status, department, crude mortality, culture confirmed HAI, HAI related mortality, duration of hospital stay, total cost and most common primary diagnosis during ICU care

Variables	NICU	PICU	SICU	Crude mortality	HAI*	HAI mortality	Total	Hospital stay (days)	Cost
Female (%)	117 (40)	82 (28)	91 (31)	73 (25)	18 (6)	10 (3.5)	290	16	1909
Male (%)	206 (51)	88 (22)	108 (27)	94 (23)	39 (10)	16 (4)	402	19	2311
CRE + at admission (%)	**151 (49)**	**84 (27)**	**71 (23)**	**65 (21)**	**30 (10)**	**15 (5)**	**306**	**24.2**	**2419**
CRE—at admission (%)	172 (44)	88 (23)	128 (33)	102 (26)	29 (7)	11 (3)	388	14.2	1917
CRE Acquisition (%)	**82 (56)**	**39 (27)**	**26 (18)**	**19 (13)**	**20 (14)**	**5 (3)**	**147**	**24.3**	**2852**
No CRE acquisition (%)	**27 (28)**	**21 (22)**	**49 (51)**	**21 (28)**	**2 (2)**	**1 (1)**	**97**	**14.6**	**2295**
CRE—not re-screened** (%)	63 (44)	28 (19)	53 (37)	63 (44)	7 (5)	5 (3.5)	144	3.8	712
CRE + total (%)	**233 (51)**	**123 (27)**	**97 (21)**	**84 (19) A**	**50 (11) B**	**20 (4) C**	**453 T**	**23.4 D**	**2568 E**
CRE—total (%)	**90 (37)**	**49 (20)**	**102 (42)**	**83 (34) A**	**9 (4) B**	**6 (2.5) C**	**241 T**	**8.1 D**	**1351 E**
Total (%)	323 (46)	172 (25)	199 (29)	167 (24)	59 (8.5)	26 (3.7)	694	18.0	2141
Age at screening (months)	0.32	22.2	32.6	13.1	3.88	1.16	12.9	NA	NA
Pneumonia + bronchiolitis (J12–21)	44	65	18	14 (11)	11 (9)	1 (1)	127	20	2344
Congenital malformations (Q01–99)	25	11	59	24 (25)	8 (8)	3 (3)	95	20	2303
Extreme immaturity + Other preterm infants (P07–07.3)	51	3	0	13 (24)	8 (15)	1 (2)	54	19	2644
Bacterial sepsis of newborn (P36)	45	0	0	18 (40)	9 (20)	6 (13)	45	20	2006
Birth asphyxia + Respiratory distress (P21–P22.1)	38	0	2	10 (25)	5 (13)	5 (13)	40	14	1531
Malignant neoplasms (C22–C92)	1	6	28	8 (23)	1 (3)	0	35	18	1707
Neonatal jaundice (P55.0–P59.0)	23	1	1	0	0	0	25	16	701
Sepsis + Septic shock (A41.0–41.9 + R57.2)	1	16	4	12 (57)	1 (5)	1 (5)	21	25	3303
Intracerebral haemorrhage (I61)	1	0	18	5 (26)	0	0	19	13	1040
Acuter respiratory failure (J96.0)	2	7	3	10 (83)	0	0	12	12	4035

^*^Culture confirmed HAI caused by *Enterobacteriaceae*

^**^CREneg at admission and not re-screened and discharged < 1 week

CREpos total vs CREneg total:

A. Crude mortality vs Total mortality, significant *p* < 0.001 Chi^2^

B. HAI (n), significant *p* < 0.002 Chi^2^

C. HAI mortality vs Crude mortality, significant *p* < 0.005 Chi^2^

D. Hospital stay days, significant *p* < 0.001 t-test

E. Cost, significant *p* < 0.001 t-test

### CRE acquisition

**Fig. 3:**
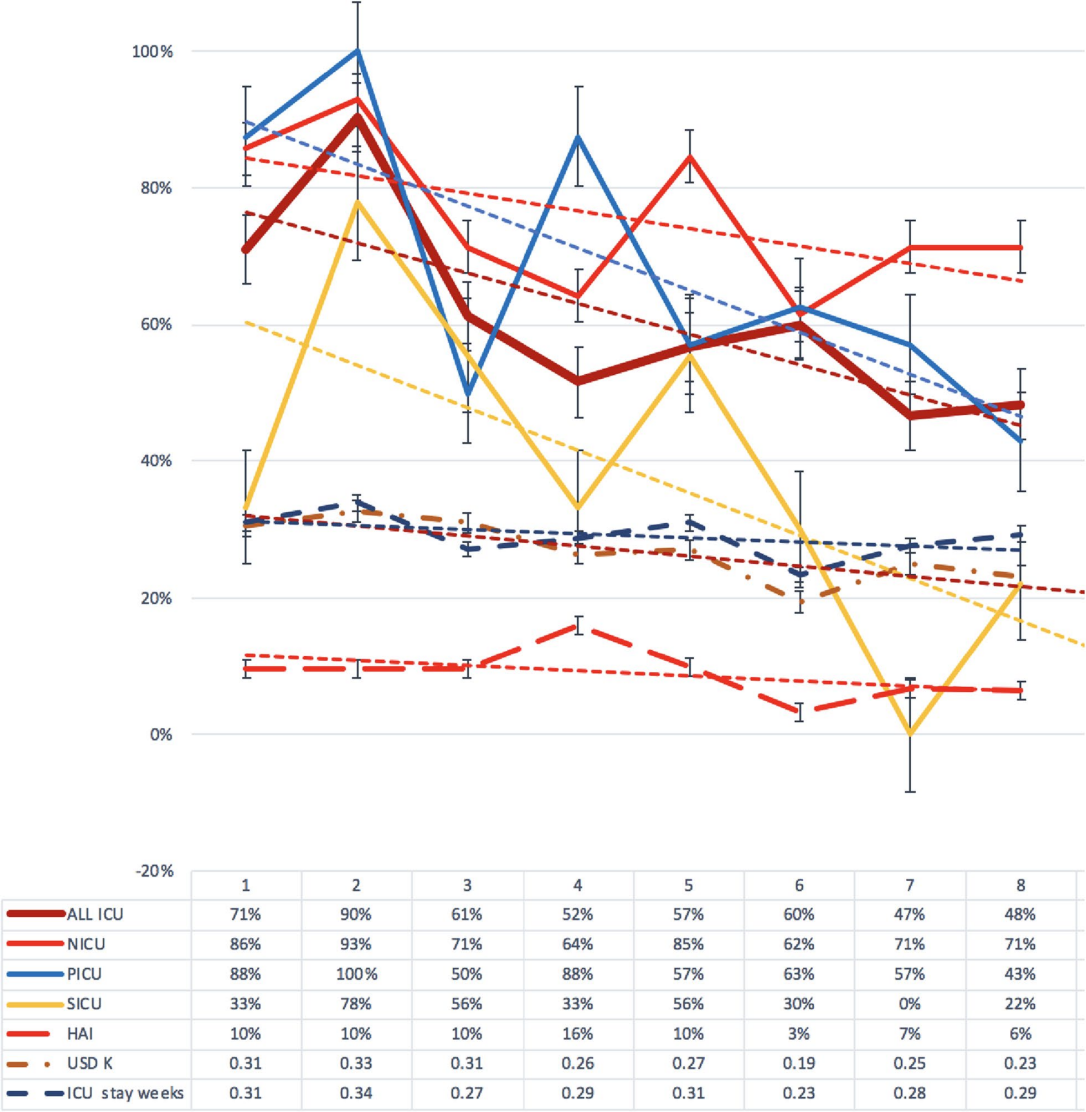
Change per period in CRE acquisition rate, culture confirmed HAI, healthcare costs in USD (0.1 = 1000 USD) and average duration of hospital stay (0.1 = 1 week): per period and linear trend (dashed strait line) and standard error for each variable and period (vertical bar)

244 patients CREneg at admission were rescreened. An average 2.35 screenings were performed per patient, with 147 (60%) CREpos in rescreening (acquisition). Patients with CRE acquisition vs. no acquisition were screened on average 2.5 and 2.1 times, respectively. The cohort of 244 CREneg patients were stratified according to ICU: NICU (109), PICU (60) and SICU (75). In each ICU, all patients were split in 8 similar size groups, (periods), based on time of admission, roughly corresponding to weeks. A significant negative correlation between CRE acquisition and periods was seen (*p* < 0.0005), corresponding to a significant decrease in acquisition in univariate- and multivariate analysis (OR − 3.2, *p* < 0.005). A significant decrease of CRE acquisition was seen from the 2nd to the 8th period in the PICU (*p* < 0.05) and SICU (*p* < 0.005), however not in the NICU (*p* < 0.2). When assessing CRE acquisition in NICU from the 2nd up to the 6th period (n = 68), a significant (*p* < 0.05) decrease of CRE acquisition was seen (Fig. 3).

CRE acquisition was significantly correlated with culture confirmed HAI (*p* < 0.005), weeks of hospital stay (*p* < 0.0005) and total treatment cost (*p* < 0.05), but not with mortality and bacterial infections based on CD10 codes (Table 3). In multivariate analysis, a significant correlation between CRE acquisition and weeks of hospital stay was seen (*p* < 0.001), however not with healthcare cost, the latter likely due to highly significant (*p* < 0.00001) co-variation between healthcare cost and weeks of hospital stay. A significant correlation (*p* < 0.001) was seen between periods and decreased healthcare cost (cost decreased with time), but no significant correlation with HAI or weeks of hospital stay.

**Table 3:** Uni-and multi-variate analysis (Pearson correlation and multiple linear regression) based on CRE acquisition vs. no acquisition in relation to time (periods)

			Uni-variate			Multi-variate			
Variable	CRE Acquisition	No CRE Acquisition	Pearson *p*-value	CI 95% lower	CI 95% upper	S.D	Odds ratio	2-tail *p*-value	1-tail *p*-value
PERIOD	149 (61%)	95 (39%)	< 0.0005	− 0.33	− 0.09	0.0123	− 3.19	0.0016	0.0008
HAI	20 (14%)	2 (2%)	< 0.005	0.07	0.31	0.1031	2.73	0.0067	0.0034
Mortality	19 (13%)	20 (21%)	0.12 NS	− 0.24	0.006				
Infection	49 (33%)	21 (22%)	0.13 NS	− 0.004	0.24				
USD	2855	2287	< 0.05	− 0.012	0.236	0.0197	− 1.22	0.22 NS	0.11 NS
WEEKS*	3.26	2.37	< 0.0005	0.109	0.347	0.0226	3.54	0.00048	0.00024
NICU	82 (75%)	27 (25%)	3.66 NS	0.14	0.37				
PICU	39 (65%)	21 (35%)	0.47 NS	− 0.08	0.17				
SICU	28 (37%)	47 (63%)	2.23 NS	− 0.43	− 0.21				

HAI = culture confirmed HAI, Infection = bacterial infection ICD10 diagnosis, USD = treatment cost, WEEKS* = duration of hospital stay in weeks

### Microbiological findings

The most common colonizing CRE were *E. coli* (41% of patients and 35% of isolates), *K. pneumoniae* (38% of patients and 32% of isolates), *KESC (Klebsiella spp., Enterobacter spp., Serratia marscescens, and Citrobacter spp.)* (26% of patients and 22% of isolates) and “finally” other G-bacteria such as *Acinetobacter baumannii* and *Pseudomonas aeruginosa* (14% of patients and 12% of isolates). In culture confirmed HAI, *K. pneumoniae* (59%) and *E. coli* (33%) were the most common (Table 4). Several patients had growth of more than one bacteria in cultures. Repeated cultures with same strain form the same patient was excluded from the data set.

**Table 4:** Bacterial growth from CRE screenings and culture confirmed HAI (N = 694 patients)

	*E. coli* N (%)	*K. pneumonia* N (%)	*KESC #* N (%)	*Other##* N (%)	Total N isolates N (%)	Mortality N (%)
CREpos screening (%)	185 (41)P 185 (35)I	173 (38)P 173 (32)I	116 (26)P 116 (22)I	62 (14)P 62 (12)I	453 (118) P* 536 (100) I *	167/694(24)
HAI isolates (%)	21/59 (36)	37/59 (63)	1/59(2)	0 (0)	59/59 (100)	26/59 (44)
Blood (%)	7/21 (33)	15/37(41)	*7/17 (41)*	*0 (0)*	22/59 (37)	10/22 (45)
Tracheal aspirate (%)	14/21 (66)	22/37 (59)	*10/17 (59)*	*3/3 (100)*	37/59 (63)	16/37 (43)
Carbapenem R/S (%R)	6/4(60)	15/3(83)	*7/0 (100)*	*2/2 (100)*	22/7 (76)	R12/22(55); S3/7(43)
Gentamycin R/S (%R)	6/4 (60)	13/5 (72)	*7/0 (100)*	*2/2 (100)*	20/9 (69)	R11/20(55); S4/9(44)
Mortality CREpos (%)	36/185 (19)	34/173 (20)	*20/116 (17)*	*10/62 (16)*	71/453 (16)	71/694 (10)
Mortality HAI (%)	8/21 (38)	17/37(46)	*8/17 (47)*	*3/3 (100)*	26/59 (44)	26/694 (4)

R = resistant, S = sensitive. E*C* = *Escherichia coli, KP* = *Klebsiella pneumoniae*, HAI = Culture Confirmed HAI

P = patients (n = 453), I = isolates (N = 534) *In average 1,18 isolates per screening culture (534/453)

^#^KESC (*Klebsiella spp., Enterobacter spp., Serratia marscescens,* and *Citrobacter spp*.)

^*##*^ Other including *Pseudomonas Aeruginosa* and *Acinetobacter baumannii*

Italicized area CC HAI isolates among screening positive patients (CC both *Escherichia coli*, *Klebsiella pneumoniae CC and Enterobacter*)

### Hospital acquired infections

Of the 694 patients screened for CRE, 59 (8.5%) acquired culture confirmed HAI caused by *Enterobacteriaceae* (Table 2). *K. pneumoniae* HAI was the most common: (n = 37), with 15 cultures from blood and 22 from tracheal aspirate. Antibiotic susceptibility data was available for 18 cultures, 15 (83%) carbapenem resistant and 13 (72%) gentamycin resistant. HAI with *E. coli*: 21 cultures, 7 blood and 14 tracheal aspirate, antibiotic susceptibility data available for 10 cultures, 6 (60%) resistant to *both* carbapenem and gentamycin (Table 4). Patients with culture confirmed HAI with *Enterobacteriaceae* when compared to all other patients, had significantly higher mortality (*p* < 0.001), 32% vs. 13%; CRE colonization (*p* = 0.016), 62% vs. 50%; CRE acquisition (*p* = 0.0078), 85% vs. 57%; longer hospital stay (*p* = 0.012), 23.2 days vs. 17.7 days and costs (*p* = 0.029), 2876 USD vs. 2116 USD (Table 2).

## Discussion

### Main findings

In all ICU’s combined, CRE acquisition decreased significantly from 90% in period 2 to 48% in period 8 during the intervention. The decrease was most pronounced in the PICU and SICU: from 100 and 78% period 2, to 43% and 22% period 8, respectively. In the NICU, a significant decrease of CRE acquisition was detected from period 2 (93%) to period 6 (62%), however an increase to period 8 (71%). The NICU has the most beds per room, highest rate of admission, several concurrent cohorting of respiratory syncytial virus (RSV) infections, HAI and preterm neonates, and suffered from overcrowding towards the end of the study, likely explaining the increase seen in latter periods. In the SICU, the CREpos cohort was treated in an isolation room. Smaller cohorts and isolation rooms with designated personnel could further decrease the risk for CRE acquisition.

,^19^].

### Point prevalence survey result

,^7^,^7^] have shown a constant high- and increasing rate of CRE colonization in Vietnamese hospitals and ICU’s.

### CRE colonization secondary endpoints

,^4^].Colonization was associated with increased risk for HAI and mortality [7,8,3,5,9,20,21–24] This strategy has been successful in high income country hospitals [25,

,^7^,^2^,^3^,^7^,^26^]. CRE surveillance in province hospitals is needed to contain the transmission.

### HAI

Patients with CRE colonization and acquisition had significantly higher rates of culture confirmed HAI with *Enterobacteriaceae*,2). Correlation between colonization and HAI was shown in our previous study [7] and has been established in other studies also [27,25,28].

### Microbiology findings

,*E. coli*; 38% *K. pneumoniae*; 26% *KESC* and 13% for other G-negative bacteria. In our previous PPS from 2017 [7,*K. pneumoniae,* and 58.5% for *E. coli *[7]*.*,*K. pneumoniae*,*Enterobacteriaceae,* such as *Acinetobacter baumannii* and *Pseudomonas aeruginosa,* which are also an important cause of severe HAI with high mortality in Vietnamese pediatric ICU’s [8].

### Treatment outcome

The mortality rate in CRE colonized patients with culture confirmed HAI was significantly higher (24%) compared to non-colonized patients with HAI (7%). Similar results was seen in our previous 2017 study [7,7]. Other studies have also shown high mortality rates in patients with infections caused by CRE [12,20,*Enterobacteriaceae,*,

### Costs

,^9^,^23^,^29^–^31^,

The hospital cost in Vietnam is to a high extent covered by the health insurance, provided for free to children up to 6 years of age. At the time of the intervention, the costs for CRE screening were not reimbursed, despite potentially being able to save large resources for the health insurance. Patients with HAI occupy an ICU place for a longer time and blocks new admissions that require more investigations, hence pose an economic burden for the hospitals, in addition to increased patient mortality and morbidity.

The intervention was performed at a low cost, about 3 USD per agar plate, which can be used for two cultures. Costs for laboratory and ICU labor was however not accounted for. The aim of this intervention was to perform it at a cost level, which would allow a continuous CRE screening and cohort care.

### Strengths and limitations

One major limitation in this study is that it was an interventional study with no control group. Further, the intervention, for ethical reasons, started immediately after a patients CRE status was revealed. As this was no randomized controlled trial of CRE screening and cohort care, the results of this study can only be interpreted as indicative. The intervention was performed in pre-existing facilities and without extra staff, beside the research team. The cohorts could in most situations not be completely physically separated and it was not always possible to have designated staff. The high CRE colonization rate at admission made it challenging to isolate patients. Of 604 patients screened CREneg at admission, only 244 (40.4%) were re-screened. Reasons for this include that many patients were admitted for < 48 h, patients were moved with short notice when discharged from ICU, lack of staff and reluctance to rectal swab patients after death. Despite these limitations, which in LMIC are a realistic actual setting, our study showed that admission CRE screening and cohort care intervention significantly *can* reduce the rates of CRE acquisition and HAI as well as shorten hospital stays and decrease costs.

Higher levels of HAI, higher treatment costs and a higher level of HAI-related mortality was seen in CREpos patients when compared to CREneg. One possible confounder is whether CREpos patients represents a group of patients with higher levels of co-morbidities (more ill patients) and therefore more prone to get colonized, have longer treatment periods in the hospital and be more likely to get nosocomial infections (HAI’s) and suffer a mortal outcome from these. The intervention design without a control group does not allow for determining whether associations of CRE colonization (initial) and acquisition with HAI, costs, and mortality (later), are causal. The group of patients treated in a separate cohort while awaiting their admission CRE screening results contained both CREneg and CREpos patients. Although it was the ambition to distance the patients in the group, possibly exposure could not be excluded, providing a risk of patients initially screening CREneg becoming colonized while awaiting their screening results. Some patients were likely colonized during this waiting period, which was deemed non-preventable in this intervention. In ideal conditions, complete isolation of patients from one another would be preferred but was not possible within the VNCH ICU’s. The 7-day follow-up screening on all admitted patients was used, to limit the transmission of CRE in the CREneg cohort.

## Conclusion

The admission CRE screening and cohort care intervention showed that CRE acquisition can be reduced with limited resources and be cost-effective by reducing CRE colonization, HAI and duration of hospital stay. As large proportions of patients were CRE colonized at admission, indicating a significant CRE spread in non-tertiary level hospitals, CRE screening should be implemented in all healthcare levels in the endemic Vietnamese system, however likely not applicable to other countries with lower levels of CRE. To enable permanent CRE admission screening and cohort care implementation, economic incentives for the health care sector to use CRE screening as a tool (such as reimbursement for screening costs from the health insurance system) should be warranted. The significant reduction in CRE acquisition found in this study is indicative, as there was no control group and further since the intervention, for ethical reasons, started as soon as patients CRE status were known. To create evidence, a randomized controlled trial in a larger patient material assessing the effects of CRE screening and cohort care would be recommended.

## Data Availability

All data is stored in a secure place with all patient information unidentifiable, for patient security reasons. All data can be accessed only after written permission to the hospital and Mattias.Larsson@ki.se.

## References

[1] World Health Organization. Antimicrobial resistance: global report on surveillance2014. 1 online resource (xxii, 232 pages) p.

[2] Kiratisin P, Chongthaleong A, Tan TY, Lagamayo E, Roberts S, Garcia J (2012). Comparative in vitro activity of carbapenems against major Gram-negative pathogens: results of Asia-Pacific surveillance from the COMPACT II study. Int J Antimicrob Agents.

[3] Nordmann P, Dortet L, Poirel L (2012). Carbapenem resistance in Enterobacteriaceae: here is the storm!. Trends Mol Med.

[4] El-Gamal MI, Brahim I, Hisham N, Aladdin R, Mohammed H, Bahaaeldin A (2017). Recent updates of carbapenem antibiotics. Eur J Med Chem.

[5] Martin A, Fahrbach K, Zhao Q, Lodise T. Association between Carbapenem resistance and mortality among adult, hospitalized patients with serious infections due to enterobacteriaceae: results of a systematic literature review and meta-analysis. 2018 (2328–8957 (Print)). eng. 10.1093/ofid/ofy150 PMC6054228 30046639

[6] Bratu S, Tolaney P, Karumudi U, Quale J, Mooty M, Nichani S (2005). Carbapenemase-producing Klebsiella pneumoniae in Brooklyn, NY: molecular epidemiology and in vitro activity of polymyxin B and other agents. J Antimicrob Chemother.

[7] Tran DM, Larsson M, Olson L, Hoang NTB, Le NK, Khu DTK (2019). High prevalence of colonisation with carbapenem-resistant Enterobacteriaceae among patients admitted to Vietnamese hospitals: risk factors and burden of disease. J Infect.

[8] Peters L, Olson L, Khu DTK, Linnros S, Le NK, Hanberger H, et al. Multiple antibiotic resistance as a risk factor for mortality and prolonged hospital stay: a cohort study among neonatal intensive care patients with hospital-acquired infections caused by gram-negative bacteria in Vietnam. PLoS ONE. 2019;14(5):e0215666. 10.1371/journal.pone.0215666 PMC6505890 31067232

[9] Stewardson AJ, Marimuthu K, Sengupta S, Allignol A, El-Bouseary M, Carvalho MJ (2019). Effect of carbapenem resistance on outcomes of bloodstream infection caused by Enterobacteriaceae in low-income and middle-income countries (PANORAMA): a multinational prospective cohort study. Lancet Infect Dis.

[10] Tumbarello M, Viale P, Viscoli C, Trecarichi EM, Tumietto F, Marchese A (2012). Predictors of mortality in bloodstream infections caused by Klebsiella pneumoniae carbapenemase-producing K. pneumoniae: importance of combination therapy. Clin Infect Dis.

[11] Kang JS, Yi J, Ko MK, Lee SO, Lee JE, Kim KH (2019). Prevalence and risk factors of carbapenem-resistant enterobacteriaceae acquisition in an emergency intensive care unit in a tertiary hospital in Korea: a case-control study. J Korean Med Sci.

[12] Tomczyk S, Zanichelli V, Grayson ML, Twyman A, Abbas M, Pires D (2019). Control of carbapenem-resistant enterobacteriaceae, acinetobacter baumannii, and pseudomonas aeruginosa in healthcare facilities: a systematic review and reanalysis of quasi-experimental studies. Clin Infect Dis.

[13] DalBen MF, Teixeira Mendes E, Moura ML, Abdel Rahman D, Peixoto D, Alves Dos Santos S, et al. A model-based strategy to control the spread of carbapenem-resistant enterobacteriaceae: simulate and implement. Infect Control Hosp Epidemiol. 2016;37(11):1315–22. 10.1017/ice.2016.168 27609341

[14] Richter SS, Marchaim D. Screening for carbapenem-resistant Enterobacteriaceae: Who, when, and how? Virulence. 2017;8(4):417–26. 10.1080/21505594.2016.1255381 PMC5477693 27813699

[15] Ambretti SA-OX, Bassetti M, Clerici P, Petrosillo N, Tumietto F, Viale P, et al. Screening for carriage of carbapenem-resistant Enterobacteriaceae in settings of high endemicity: a position paper from an Italian working group on CRE infections. 2019 (2047–2994 (Electronic)). eng. 10.1186/s13756-019-0591-6 PMC6693230 31423299

[16] Biomerieux. CHROMID® CARBA SMART Agar 2020 [cited 2020 9 sep]. CHROMID CARBA SMART Agar description]. Available from: https://www.biomerieux-diagnostics.com/chromid-carba-smart-agar.

[17] Wessa P. Free Statistics Software. In: Education OfRDa, editor. version 1.2.1 ed: wessa.net; 2020.

[18] J. S. socscistatistics 2020. Available from: https://www.socscistatistics.com/.

[19] Schwaber MJ, Lev B, Israeli A, Solter E, Smollan G, Rubinovitch B (2011). Containment of a country-wide outbreak of carbapenem-resistant Klebsiella pneumoniae in Israeli hospitals via a nationally implemented intervention. Clin Infect Dis.

[20] Chiotos K, Tamma PD, Flett KB, Karandikar MV, Nemati K, Bilker WB, et al. Increased 30-day mortality associated with carbapenem-resistant enterobacteriaceae in children. Open Forum Infect Dis. 2018;5(10):ofy222. 10.1093/ofid/ofy222 PMC6186173 30338267

[21] Centers for Disease Control and Prevention. Facility guidance for control of carbapenemresistant Enterobacteriaceae (CRE): November 2015 update - CRE toolkit 2015. Atlanta. 2015.

[22] Magiorakos AP, Burns K, Rodriguez Bano J, Borg M, Daikos G, Dumpis U, et al. Infection prevention and control measures and tools for the prevention of entry of carbapenem-resistant Enterobacteriaceae into healthcare settings: guidance from the European Centre for Disease Prevention and Control. Antimicrob Resist Infect Control. 2017;6:113. 10.1186/s13756-017-0259-z PMC5686856 29163939

[23] Prevention. CfDCa. Facility guidance for control of Carbapenem-resistant Enterobacteriaceae (CRE): November 2015 update - CRE toolkit United States Centers for Disease Control and Prevention; 2015. p. 22.

[24] World Health Organization. Guidelines for the Prevention and Control of Carbapenem-Resistant Enterobacteriaceae, Acinetobacter baumannii and Pseudomonas aeruginosa in Health Care Facilities. Guidelines for the Prevention and Control of Carbapenem-Resistant Enterobacteriaceae, Acinetobacter baumannii and Pseudomonas aeruginosa in Health Care Facilities. WHO Guidelines Approved by the Guidelines Review Committee. Geneva2017.

[25] Dickstein Y, Edelman R, Dror T, Hussein K, Bar-Lavie Y, Paul M (2016). Carbapenem-resistant Enterobacteriaceae colonization and infection in critically ill patients: a retrospective matched cohort comparison with non-carriers. J Hosp Infect.

[26] de la Serna A, Pascual A, GUTIERREZ B, al. e. EURECA, Study 2019 [cited 2019 2019/12/31]. Study NONCT02709408 Study NameEURECA Study Type Observational Study Status Recruitment Complete Enrollment Period 05/16 - 04/18; European prospective cohort study on Enterobacteriaceae Showing resistance to carbapenems (EURECA)]. Available from: https://www.combacte.com/trials/eureca/.

[27] Giannini MA, Gilliam C, Owings A, Glover B, Gipson M, Hakim H (2017). Does colonization with Carbapenem-resistant *Enterobacteriaceae* correlate to infection?. Am J Infect Control.

[28] Tischendorf J, de Avila RA, Safdar N. Risk of infection following colonization with carbapenem-resistant Enterobactericeae: a systematic review. Am J Infect Control. 2016;44(5):539–43. 10.1016/j.ajic.2015.12.005 PMC5262497 26899297

[29] Centers for Disease Control and Prevention. The Direct Medical Costs of Healthcare-Associated Infections in U.S. Hospitals and the Benefits of Prevention. internet: Centers for Disease Control and Prevention,; 2009 [cited 2020 7 oct]. Available from: http://www.cdc.gov/hai/pdfs/hai/scott_costpaper.pdf. .

[30] Hughes JM (1988). Study on the efficacy of nosocomial infection control (SENIC Project): results and implications for the future. Chemotherapy.

[31] Umscheid CA, Mitchell Md Fau - Doshi JA, Doshi Ja Fau - Agarwal R, Agarwal R Fau - Williams K, Williams K Fau - Brennan PJ, Brennan PJ. Estimating the proportion of healthcare-associated infections that are reasonably preventable and the related mortality and costs. Infect Control Hosp Epidemiol. 2011;32(2)(1559–6834 (Electronic)):101–14. 10.1086/657912 21460463

